# Evaluation of the impact of percutaneous coronary intervention of chronic total occlusion on regional myocardial function using strain echocardiography

**DOI:** 10.1186/s43044-019-0007-1

**Published:** 2019-09-05

**Authors:** Abd ElFattah Kholeif, Eman El sharkawy, Mohamed Loutfi, Mohamed ElGowelly

**Affiliations:** 1 Faculty of Medicine, Alexandria University, Alexandria, Egypt; 2 Alexandria, Egypt

**Keywords:** Chronic total occlusion, Percutaneous coronary intervention, Tissue Doppler strain echocardiography

## Abstract

**Background:**

Successful revascularization of chronic total occlusions has been associated with potential effects on left ventricular (LV) function. Strain and strain rate are more sensitive measures of LV mechanics than LV ejection fraction (LVEF). This study was conducted to investigate the impact of revascularization of chronic total occlusion (CTO) on LV function using tissue Doppler (TDI) strain echocardiography.

**Results:**

This study included 60 patients divided into two main groups: the percutaneous coronary intervention (PCI) group including patients who had a successful PCI of CTO on left anterior descending (LAD) artery and was presented for elective PCI with symptomatic angina and/or positive functional ischemic study. They included 18 males with a mean age of 57 ± 5 years. The optimum medical treatment (OMT) group, including 30 patients, had non-revascularized CTO-LAD and was kept on OMT alone; 20 of them were males with a mean age of 58 ± 4 years. In the PCI group, there was a significant improvement in all the TDI strain parameters of the LAD territory segments. Six months after PCI, the peak systolic strain rate improved from − 0.65 ± 0.21 to 1.05 ± 0.31 1/s (*p* value < 0.01), the peak systolic strain improved from 6.54 ± 2.48 to 11.51 ± 3.33% ( *p* value < 0.001 ), and the end systolic strain improved from 7.88 ± 2.77 to 10.51 ± 3.14% (*p* value < 0.01 ). There was no significant improvement in the mean LVEF (60.70 ± 8.33 vs 61.91 ± 8.16% (*p* value = 0.6)). In the OMT group, there was no improvement in all the strain parameters and there was no change in the mean LVEF. In the PCI group, there was a significant improvement in the angina class (*p* value = 0.03) while, in the OMT group, there was no significant improvement (*p* value = 0.835).

**Conclusions:**

Successful PCI for CTO improved regional LV myocardial function assessed by TDI strain echocardiography. This improvement was associated with improvement in the angina class.

## Background

Coronary chronic total occlusions (CTOs) are defined as an occluded coronary segment with thrombolysis in myocardial infarction (TIMI) flow 0 for ≥ 3 months duration [1]. Approximately 18–33% of patients with significant coronary disease on coronary angiography have at least one CTO [2].

Successful percutaneous coronary interventions (PCIs) of CTOs have been shown to improve the left ventricular (LV) systolic function, reduce angina, increase exercise capacity, and reduce the need for bypass surgery [3].

Two-dimensional (2D) echocardiography has been described as the ideal imaging modality for the assessment of global and regional ventricular function. However, conventional assessment of wall motion, on the basis of visual interpretation of endocardial excursion and myocardial thickening, has the limitations of being a qualitative method and is subjective and experience-dependent [4].

Tissue Doppler (TDI) imaging has been introduced in an attempt to provide a more objective assessment of myocardial contractility but is subject to the confounding effects of cardiac translational motion and passive pathological tethering [5].

These limitations may be overcome by the measurement of local myocardial deformation parameters with strain and strain rate echocardiography [6].

The aim of this study was to evaluate the impact of PCI of CTO on clinical outcome and regional myocardial function using TDI strain echocardiography.

## Methods

### Patient selection

Our study included 60 patients prospectively recruited from the period of December 2015 till January 2018. They were divided into two groups: The PCI group, including thirty patients, was presented for elective PCI of CTO on left anterior descending (LAD) artery with symptomatic angina and/or positive functional ischemic study, had successful PCI with final TIMI flow more than II, and was kept on optimum medical treatment (OMT). They included 18 males with a mean age of 57 ± 5 years. The OMT group, including thirty patients, had non-revascularized CTO and was kept on OMT alone. Twenty of them were males with a mean age of 58 ± 4 years.

CTO was defined as the presence of TIMI flow grade 0 within the occluded artery with an estimated occlusion duration of ≥ 3 months, as suggested in the Euro CTO Club consensus document [1].

We included patients having sinus rhythm and viable myocardium in the LAD artery territory artery using dobutamine stress echocardiography or myocardial perfusion imaging study.

We excluded patients having poor echocardiographic windows, severe renal impairment (creatinine clearance < 30 ml/min), emergency PCI in acute coronary syndrome, and the presence of conduction defects in the electrocardiogram.

All individuals were subjected to a full assessment of history, a thorough clinical examination, laboratory investigation, and resting 12-lead ECG.

All participants gave written informed consent, and the study was approved by the local ethics committee.

### 2D echocardiography

The echocardiography was done within 24 h before PCI and after 6 months in the PCI group, and at baseline and after 6 months in the OMT group.

2D and TDI strain echocardiography were performed on the subjects at rest in the left lateral decubitus position with synchronized electrocardiography using a commercially available system (Philips HD11, USA) equipped with a broadband S3 transducer.

The parasternal long-axis views were used to derive the M-Mode measurements of the LV end diastolic (LVEDD) and end systolic (LVESD) dimensions.

The apical views were used to derive the LV end diastolic volume (LVEDV) and LV end systolic volume (LVESV). Ejection fraction was measured by endocardial tracing using the modified Simpson method.

Cardiac TDI velocity data from the anterior, apical, and anteroseptal wall segments were recorded using apical 2-, 3-, and 4-chamber views with the wall in the middle of the sector. All datasets were acquired over three consecutive heartbeats at a high frame rate (180–200 frames/s), with an image depth of 11–14 cm. In all acquisitions, the 2D sector angle was minimized to obtain a high frame rate. The digital cine-loop raw datasets containing both gray-scale and tissue velocity imaging information were stored for off-line analysis.

The data were analyzed off-line to obtain regional myocardial velocity, strain, and strain rate imaging profiles.

### Strain and strain rate parameters

Peak systolic strain (peakε) and strain rate (SR) were determined as the maximal negative strain and SR value during ejection (between aortic valve opening and closure (AVO and AVC respectively)), but end systolic strain (εes) was measured at AVC.

We analyzed only the segments in the territory of totally occluded LAD territory.

### CTO revascularization

Baseline angiography data included the CTO territory length of occlusion, collaterals, number of diseased vessels, and baseline SYNTAX score. PCI of the CTO was performed using available techniques, such as bilateral injections; specialized hydrophilic, tapered tip, and stiff wires; and parallel wires. Procedural success was defined as the successful recanalization of CTO with a residual stenosis of less than 20% and a TIMI flow of more than 2.

Revascularization for all significant non-CTO lesions within a vessel diameter of ≥ 2.5 mm for patients with multivessel coronary artery disease was recommended.

Patients were prescribed guideline-derived OMT, including aspirin, P2Y12 receptor inhibitors (> 12 months in case of PCI), beta-blocker, calcium channel blockers, nitrate, angiotensin-converting enzyme inhibitor/angiotensin receptor blockers, and a statin, and a follow-up for 6 months was done.

### Statistical analysis

The continuous variables are reported as the mean and standard deviations (SD), and the categorical variables are expressed as percentages. Comparisons of the categorical and continuous variables between the two groups were performed using the *χ*^2^ test and an unpaired *t*-test, respectively. A value of *p* < 0.05 was considered statistically significant.

### Results

In the PCI group, only 28 patients completed the study (one patient died and one missed from the follow-up). In the OMT group, only 27 patients completed the study (two patients died and one missed from the follow-up).

Baseline characteristics are shown in Table 1, with no significant difference between the two groups with regard to demography and risk factors.

**Table 1:** Baseline characteristics

	PCI (*n* = 30)	OMT (*n* = 30)	*p* value
Age (years)	57 ± 5.1	58.8 ± 4.72	0.89
Male sex	18	20	0.4
Hypertension	23	25	0.73
Diabetes mellitus	20	18	0.23
Hypercholesterolemia	19	15	0.54
Smoking	20	21	0.81
Previous myocardial infarction	8	11	0.12
Angina class (CCS)			0.7
I/II/III	2/18/10	1/16/13	
LVEF (%)	60.70 ± 8.33	59.46 ± 7.78	0.17
Collateral grade			0.2
3/2/1/0	24/4/2/0	20/5/3/2	
Multivessel disease	8	5	0.5
SYNTAX score	19.2 ± 3.7	22.5 ± 3.5	0.07
J-CTO score	1.9 ± 0.2	2.5 ± 0.9	0.03*

*CCS* Canadian Cardiovascular Society, *LVEF* left ventricular ejection fraction, *SYNTAX* Synergy between PCI with Taxus and Cardiac Surgery, *J-score* Japanese score

* = there is a significant difference

PCI of CTO on the LAD artery was attempted in 35 patients. It was successful in 30 patients with a success rate of 85%. Complications occurred in five patients (17%): 1 patient had post-procedural bleeding, 2 patients had a hematoma, and 2 patients had a coronary dissection.

The angiographic characteristics of the CTO-PCI group are summarized in Table 2.

**Table 2:** Angiographic characteristics

	No.	%
Approach		
Antegrade	30	100
Retrograde	0	0
Lesion passaged wire		
Low penetration force wire	8	27
Intermediate to high penetration force wire	22	73
CTO technique		
Single wire	25	83
Parallel wire	5	17
Additional support		
Balloon support	20	66
No. of stents		
One	7	23
Two	15	50
More than two	8	26
Type of stent		
BMS	5	17
DES	25	83
Post dilatation		
No	5	17
Yes	25	83
TIMI flow		
TIMI III	30	100
Less than TIMI III	0	0
Complications		
Yes	5	17
No	25	83

*CTO* chronic total occlusion, *BMS* bare metal stent, *DES* drug-eluting stent, *TIMI* thrombolysis in myocardial infarction

### Left ventricular function

In the PCI group, mean LVEF before PCI was 60.70 ± 8.33 and after 6 months was 61.91 ± 8.16 with no significant improvement (*p* value = 0.6).

In the OMT group, mean LVEF was 59.46 ± 7.78 and after 6 months was 59.15 ± 7.99 with no significant difference (*p* value = 0.42).

With regard to the regional myocardial function, the strain parameters improved significantly in the PCI group, with no improvement in the OMT group, as shown in Table 3.

**Table 3:** Regional strain parameters

	PCI		OMT	
	Before	After 6 months	Before	After 6 months
	(*n* = 28)	(*n* = 28)	(*n* = 27)	(*n* = 27)
Peak sys. SR				
Mean ± SD	0.65 ± 0.21	1.05 ± 0.31	0.62 ± 0.21	0.63 ± 0.22
*p*_1_	<0.001*		0.763	
Peak sys. strain				
Mean ± SD	6.54 ± 2.48	11.51 ± 3.33	6.67 ± 2.65	6.90 ± 2.90
*p*_1_	<0.001*		0.626	
End sys. strain				
Mean ± SD	7.88 ± 2.77	10.51 ± 3.14	7.90 ± 2.87	7.63 ± 2.64
*p*_1_	<0.001*		0.079	

*Statistically significant at *p* ≤ 0.05

Global longitudinal strain was done with no significant statistical difference between both groups (*p* value = 0.35). Regarding angina, in the PCI group, there was a statistically significant improvement in the angina class (CCS) 6 months after PCI compared to the CCS before PCI (*p* value < 0.001).

In the OMT group, there was no improvement in CCS after 6 months compared to the baseline with no statistical significance (*p* value = 0.8).

## Discussion

The present study was conducted prospectively, on selected patients with the presence of viable myocardium in LAD territory. There was a slight improvement of LVEF in some patients in the PCI group but with no statistical significance in the whole group, so there was no statistically significant improvement in the LVEF measured by 2D echocardiography in both groups after 6 months follow-up. This is in line with the study of Sotomi et al. [7] who conducted a prospective observational study with consecutive 37 CTO-PCI patients. 2D echocardiography was performed before PCI, and after 1 day and 3 months of procedure, there was no change in LVEF.

In the EXPLORE trial, Henriques et al. [8] enrolled 304 patients with acute STEMI who underwent primary PCI and had concurrent CTO. They found that mean LVEF did not differ between both the groups and this was explained by the already preserved LVEF in both the groups before revascularization.

Opposite to our observations, Hoebers et al. [9] performed a weighted meta-analysis of 34 studies (including 2243 patients) and addressed the change of LVEF after successful CTO-PCI and found a significant improvement in LVEF in those with reduced baseline LVEF.

The Total Occlusion Study of Canada (TOSCA) study revealed that the baseline LV dysfunction was an independent predictor for improvement in LVEF after revascularization of CTO [10].

Another modality to assess the LV systolic dysfunction even with no regional wall motion abnormalities at rest is the strain imaging.

In our study, we aimed to assess the effect of CTO-PCI on regional myocardial function using a strain imaging TDI technique.

The regional strain parameters improved significantly in the PCI group, with no improvement in the OMT group; other studies assessed the global longitudinal strain as in Erdogan et al. [11] who enrolled a total of 129 patients with CTO who underwent revascularization. The global longitudinal strain showed a significant increase after successful revascularization. Also in Wang et al.’s study [12], LV function after percutaneous recanalization was monitored by 2D-STE in 43 patients with coronary CTO who underwent primary PCI. The global longitudinal strain was improved as early as 1 day after CTO-PCI.

Health status and quality of life indices are increasingly used in cardiovascular studies and reflect the increasing importance of these patient-perspective endpoints as an indication for therapy.

In our study, there was a significant improvement in CCS in the PCI group with no improvement in the OMT group, and this is in line with Olivari et al. [13] who enrolled 419 consecutive patients scheduled for PCI of CTO of ≥ 30 days of duration; 88.7% of the patients had no angina symptoms, and 73.8% of those performing an exercise test were able to reach the maximum heart rate for age. Kirk Christensen et al.’s study [14] included 503 patients who underwent CTO-PCI, and after a follow-up for 3 months, there was an improvement in the CCS; 87% were in CCS before the index procedure vs. 22% during the follow-up.

There are several limitations to this study. The main limitation is its observational nature and the fact that it was non-randomized. Besides, the use of a single modality for the assessment of regional myocardial function and the limitations of TDI strain echocardiography constitute other limitations.

## Conclusion

We conclude that successful PCI for CTO, with evidence of a viable myocardium, improves LV function assessed by TDI strain echocardiography. This improvement is associated with improvement in the CCS.

## Data Availability

All the data and materials will be available on request from the corresponding author.

## Abbreviations

2D: Two dimensional
BMS: Bare metal stent
CTO: Chronic total occlusion
DES: Drug-eluting stent
LAD: Left anterior descending artery
LV: Left ventricle
LVEDD: Left ventricular end diastolic dimension
LVEDV: Left ventricular end diastolic volume
LVEF: Left ventricular ejection fraction
LVESD: Left ventricular end systolic dimension
LVESV: Left ventricular end systolic volume
OMT: Optimum medical treatment
PCI: Percutaneous coronary intervention
SR: Strain rate
STEMI: ST segment elevation myocardial infarction
TDI: Tissue Doppler imaging
